# Understanding the molecular aspects of oriental obesity pattern differentiation using DNA microarray

**DOI:** 10.1186/s12967-015-0692-9

**Published:** 2015-10-19

**Authors:** Sun Woo Hong, Jae-Wook Yoo, Shambhunath Bose, Jung-Hyun Park, Kyungsun Han, Soyoun Kim, Chi-Yeon Lim, Hojun Kim, Dong-ki Lee

**Affiliations:** Global Research Laboratory for RNAi Medicine, Department of Chemistry, Sungkyunkwan University, Suwon, Gyeonggi-do 440-746 Republic of Korea; Institute of Basic Science, Sungkyunkwan University, Suwon, Korea; College of Pharmacy, Dongguk University, Seoul, Korea; Department of Oriental Rehabilitation Medicine, Graduate School of Oriental Medicine, Dongguk University-Seoul, 814 Siksa, Goyang, Gyeonggi 410-773 Republic of Korea; Department of Biomedical Engineering, Dongguk University, Seoul, Korea; Department of Medicine, Graduate School, Dongguk University Seoul, Seoul, Korea

**Keywords:** Microarray, Oriental medicine, Pattern identification, Obesity

## Abstract

**Background:**

Human constitution, the fundamental basis of oriental medicine, is categorized into different patterns for a particular disease according to the physical, physiological, and clinical characteristics of the individuals. Obesity, a condition of metabolic disorder, is classified according to six patterns in oriental medicine, as follows: spleen deficiency syndrome, phlegm fluid syndrome, yang deficiency syndrome (YDS), food accumulation syndrome (FAS), liver depression syndrome (LDS), and blood stasis syndrome. In oriental medicine, identification of the disease pattern for individual obese patients is performed on the basis of differentiation in obesity syndrome index and, accordingly, personalized treatment is provided to the patients. The aim of the current study was to understand the obesity patterns in oriental medicine from the genomic point of view via determining the gene expression signature of obese patients using peripheral blood mononuclear cells as the samples.

**Methods:**

The study was conducted in 23 South Korean obese subjects (19 female and four male) with BMI ≥25 kg/m^2^. Identification of oriental obesity pattern was based on the software-guided evaluation of the responses of the subjects to a questionnaire developed by the Korean Institute of Oriental Medicine. The expression profiles of genes were determined using DNA microarray and the level of transcription of genes of interest was further evaluated using quantitative real-time PCR (qRT-PCR).

**Results and conclusion:**

Gene clustering analysis of the microarray data from the FAS, LDS, and YDS subjects exhibited disease pattern-specific upregulation of expression of several genes in a particular cluster. Further analysis of transcription of selected genes using qRT-PCR led to identification of specific genes, including prostaglandin endoperoxide synthase 2, G0/G1 switch 2, carcinoembryonic antigen-related cell adhesion molecule 3, cystein-serine-rich nuclear protein 1, and interleukin 8 receptor, alpha which were highly expressed in LDS obesity constitution. Our current study can be considered as a valuable contribution to the understanding of possible explanation for obesity pattern differentiation in oriental medicine. Further studies can address a novel possibility that the genomic and oriental empirical approaches can be combined and implemented in systematic and synergistic development of personalized medicine.

This clinical trial was registered in Clinical Research Information Service of Korea National Institute of Health (https://cris.nih.go.kr/cris/index.jsp). Registration number: KCT0000387

**Electronic supplementary material:**

The online version of this article (doi:10.1186/s12967-015-0692-9) contains supplementary material, which is available to authorized users.

## Background

In recent years, DNA microarray, one of the most advanced techniques for profiling global gene expression, has emerged as a popular tool for investigation of human diseases as it enables comparison of gene expression patterns between normal and pathological tissues and provides unique data about disease mechanisms, regulatory pathways, and gene functions [1–5].

Obesity is a kind of pathophysiological condition characterized by excess accumulation of adipose tissue in the body, which is determined by genetic factors, overeating, inadequate physical activity, and improper lifestyle [6–8]. Obesity is also known to be a prime factor for increasing the risk of other serious diseases, such as diabetes, hypercholesterolemia, fatty liver disease, hypertension, arteriosclerosis, angina, cardiac infarction, stroke, Pickwick syndrome, and gout [9]. Because of these facts, the mortality of obese patients is much higher than that of normal individuals [10–13]. In parallel with the current global scenario, obesity has become one of the most life-threatening problems in South Korea. The Korea National Health and Nutrition Examination reported that the overall prevalence of Korean adult obesity was 30.7 % in 2008, compared with 21.8 % in 1998 [14].

Traditional medicine, which includes herbal medicine, acupuncture, and other non-medication therapies, represents a comprehensive system of medicine characterized by its inherent theoretical concept and practical experience [15]. Because of its unique prototype and remarkable efficacy with fewer adverse side effects, this system of medicine has gained a considerable interest internationally. Oriental medicine, which is closely related to traditional Chinese medicine, focuses primarily on constitutional approaches [15]. The term ‘body constitution’ (BC) is coined from the Chinese medicinal literature and culture to state one’s current health status which reflects the condition and function of his/her body [16]. More specifically, each constitution is categorized by the characteristics of body shape, face, voice, and psychological and physiological aspects [17, 18], and those characteristics differ between and among the constitutional types [19]. It has been stated that individuals representing different constitution types differ in their susceptibility to disease with a varied degree [20]. It has been also described that in general, a normal or balanced constitution represents an overall healthy state of an individual, whereas individuals with unbalanced constitutions are more susceptible to certain diseases [20, 21]. People can be classified into different types of body characteristics such as strong, weak, ulcer, and bleeding types based on one’s risk or tendency of disease development [22]; and it has been opined that the relative risk of several chronic diseases differs across different constitution groups [18, 23]. In other words, human constitutions can be categorized into different patterns for a particular disease depending on the physical, physiological, and clinical characteristics of the individuals. Thus, in oriental medicine, subjects with disease are diagnosed based on the pattern identification [10], a comprehensive system characterized by its own theoretical basis and practical experience [24]. More specifically, the extensive analyses of symptoms and signs performed through this unique system have implications in determining the cause, nature, and location of the illness and the patient’s physical condition, and their treatment approaches [15]. In other words, pattern identification is used as a tool by the medical professionals of oriental medicine to diagnose patients, which ultimately allows personalized treatments according to the symptoms of individual patients.

In oriental medicine, obesity is considered a disease state of humans, who are categorized as ‘Obese People,’ Soft Extratic People,’ or ‘Flesh People.’ [25] Six disease patterns in obesity among the Korean population have been identified and implemented by the Korea Institute of Oriental Medicine (KIOM, Daejeon, South Korea) [26]. These are categorized as spleen deficiency syndrome (SDS), phlegm fluid syndrome (PFS); yang deficiency syndrome (YDS); food accumulation syndrome (FAS), also termed as indigestion or food stagnation syndrome; liver depression syndrome (LDS) or stagnation of liver qi syndrome; and blood stasis syndrome (BSS) [15]. Doctors practicing oriental medicine make a diagnosis of obese patients on the basis of differentiation in obesity syndrome index and accordingly identify the disease pattern and prescribe medicines, providing personalized treatment to the patients. In the field of western medicine, in general, individual patterns are not considered in diagnosis of diseases and prescription of medicines to patients. In contrast, the oriental medicine field follows inherent or empirical traits of patients, sometimes resulting in better medicinal impact with fewer adverse side effects [26–28].

In the current study, we attempted to evaluate the oriental obesity patterns from the genomic point of view via determining the expression profiles of genes using DNA microarray and quantitative real-time PCR (qRT-PCR) techniques. Microarray-based techniques have been adopted previously for identification of the pattern of gene expression in traditional medicinal constitutions [29, 30]. In our study, gene clustering analysis of the microarray data of subjects representing FAS, LDS, and YDS patterns exhibited enriched expression profile of several genes. In addition, subsequent analysis of transcription of selected genes using qRT-PCR led to identification of marker genes that are highly expressed in LDS obesity constitution. Although our study did not categorize all oriental obesity patterns from the genomics point of view, it made a valuable contribution to the classification and acquisition of specific marker genes signifying an empirical science based oriental obesity pattern.

## Methods

### Subjects

The scientific investigation reported here was an additional study of a clinical trial conducted in Dongguk University Oriental Hospital, South Korea [registered in Clinical Research Information Service of Korea National Institute of Health, (https://cris.nih.go.kr/cris/index.jsp), registration number: KCT0000387]. Briefly, 166 obese subjects were enrolled for the main trial who responded to an advertisement that explained the purpose of the study. They were provided with a brief outline of the trial along with the exclusion criteria. The recruitment procedure was continued for more than a year (October, 2009 to November, 2010). From the entire pool of the participants, 23 subjects (19 females and 4 males) were randomly chosen for the present microarray study. Additional obese subjects were also selectively or randomly chosen from the volunteer pool to provide samples for comparative gene expression analysis. To comply with this study, subjects should be ethnically Korean and within 18 to 65-years-of-age. They should be non-smoker and must possess in-general good physical condition with BMI ≥25 kg/m^2^. Their body weight should not have changed within ±3 kg during the last 3 months. Prior to the trial, all screened volunteers were informed in detail of the purpose and principles of the study, and the freedom to withdraw from the study. The subjects were then instructed to peruse the consent forms provided to them and sign the forms if they agreed to participate in the trial. Following this, the general health conditions of the volunteers were evaluated by a physician. The following abnormal health conditions were considered among the exclusion criteria: heart, renal, or hepatic disease, history of alanine aminotransferase (ALT) or aspartate aminotransferase (AST) enzyme activity >2.5 times the normal upper limit, or level of creatinine >2.0 mg/dL, as well as malignancy. In addition, subjects under medications that may interfere with the body composition and metabolism, and subjects who had exhibited a hypersensitive reaction to a drug were excluded. Pregnancy at any time point during the study period was also among the exclusion criteria. The trial was initiated following the approval of the medical, scientific, and ethical aspects of the study by the Institutional Review Board of Dongguk University Oriental Hospital (09-CR-12).

### Identification of oriental obesity pattern

A computer-based self-reporting online questionnaire developed by Korean Institute of Oriental Medicine (KIOM, Daejeon, South Korea) was used for identification of oriental obesity pattern of the subjects (http://ammrc.kiom.re.kr/renewal/obesity/). During their first visit to the hospital, the subjects were asked to enter their responses to the queries depicted in the online form. The software program developed by KIOM analyzes the responses in the questionnaire and based on these designates every subject as one out of existing six obesity patterns in oriental medicine, as follows: SDS, PFS, YDS, FAS, LDS, and BSS. This questionnaire consists of 52 questions: 8 questions about general or systemic symptoms; 8 on personality and emotion, 18 on digestive function and 18 on circulatory function (see Additional file 1: Table S1 for further details). Each question which has different weight according to its significance for pattern diagnosis is valued on a 5-point scale (1, little; 2, mild; 3, moderate; 4, intensive; 5, very intensive). The software automatically calculates the points for each question after a logical evaluation of the relative significance of the response against the function under which the question belongs. Based on the points accumulated through the subject’s responses to the questionnaire, the KIOM software computes and assigns the score for each oriental obesity pattern. Finally, each subject is designated by a pattern which obtains the highest score among the six patterns in that particular subject. This questionnaire has been validated in several studies measuring the credibility and reliability [26, 28, 31, 32]. The agreement rate between the obesity patterns diagnosed by experts in the field of obesity in oriental medicine and the pattern diagnosis generated by the KIOM questionnaire was investigated [26, 28]. The reported Cronbach alpha coefficient ranged from 0.720 to 0.848.

### Isolation of PBMCs

Shortly after their enrolment, blood was collected from the subjects following standard protocol. PBMCs were isolated from the whole heparinized blood by standard gradient centrifugation using Ficoll-Paque Plus reagent (GE Healthcare, Little Chalfont, Waukesha, WI, USA) according to the manufacturer’s instruction. Briefly, 8 mL of whole blood was diluted with phosphate-buffered saline (PBS, pH 7.4) and added carefully to the tube filled with 15 mL of Ficoll-Paque plus reagent. PBMCs were then separated into soluble interface between the upper plasma layer and lower Ficoll layer by centrifuging the samples at 400×*g* for 30 min at room temperature followed by collection of the resulting PMBC layer. The isolated PBMC fractions were washed twice by centrifugation at 100×*g* for 10 min at room temperature using PBS.

### RNA preparation and DNA microarray

Total RNA of the PMBCs was extracted using TRI reagent (Ambion, Austin, TX, USA) and the RNeasy mini kit (Qiagen, Hilden, Germany) following the reagent and kit manufacturer’s instructions, respectively. The yield of RNA ranged from 5.02 to 15.37 μg with an average of 8.85 μg. The integrity of extracted RNA was verified by gel electrophoresis. DNA microarray of the samples and subsequent analysis of the data were performed as described previously [33]. Briefly, 5 μg of total RNA was reverse transcribed for generation of double-stranded cDNA (dscDNA) using a SuperScript double-stranded cDNA synthesis kit (Invitrogen, Carlsbad, CA, USA). Reactions were terminated by addition of EDTA followed by RNase A treatment. Samples were then subjected to ethanol-precipitation and finally rehydrated to make the stock solution of dscDNA at a concentration of 250 ng/μl. Next, 1 μg dscDNA was labeled with Cy3-conjugated random 9-mer (TriLink Biotechnologies, San Diego, CA, USA) using Klenow fragment (NEB, Beverly, MA, USA); the labeled samples were then subjected to isopropanol precipitation. Four micrograms of Cy3-labeled DNA (containing sample tracking control and alignment oligo) was then hybridized to NimbleGen, 12-plex, human microarray slides (Human Gene Expression 12 × 135 K Microarray, NimbleGene, Madison, WI, USA) for 18 h at 42 °C using the NimbleGen Hybridization system (NimbleGen). Subsequently, the array slides were washed by vigorous agitation in 1 × SSC + 0.1 % SDS for 5 min at 55 °C and in 0.1 × SSC + 0.1 % SDS for 5 min at room temperature. The slides were then rinsed with distilled water and dried by centrifugation. The array images were captured using an InnoScan 900 Series Microarray Scanner (Innopsys, Carbonne, France) and the signals extracted from the scanned images were then imported into NimbleScan software (version 2.5, Nimblegen) for grid alignment and analysis of gene expression data. Expression data were normalized using a quantile normalization method [34] and Robust Multichip Average (RMA) algorithms [34]. Gene ontology (GO) analysis was performed using the software toolkit provided in the web-accessible Database for Annotation, Visualization, and Integrated Discovery (DAVID) programs (http://niaid.abcc.ncifcrf.gov/home.jsp) [35]. For this analysis, the selected gene list was uploaded into the program and the gene list of Nimblegen microarray chip was used as the background. For clustering analysis, data from microarray was uploaded to the Cluster program and cluster analysis was performed by applying parameters of interest, as described previously [36]. Clustered data were visualized using the TreeView program.

### Quantitative real-time PCR (qRT-PCR)

Total RNA of the PMBCs was extracted using TRI reagent (Ambion) according to the reagent manufacturer’s instructions; 500 ng of RNA was processed for cDNA synthesis using the high capacity cDNA reverse transcription kit (Applied Biosystems, Foster City, CA, USA) following the instructions provided by the kit manufacturer. qRT-PCR of the samples was performed in a StepOne™ real-time PCR system (Applied Biosystems). The primer sequences used in our experiment were as follows: NM_000634 forward, 5′-ACC TGG CCG GTG CTT CAG TTA G-3′ and reverse, 5′-TCC CAG CAG GCT CAG CAG GAA-3′; NM_000963 forward, 5′-TTG CCC GAC TCC CTT GGG TGT-3′ and reverse, 5′-AAG TCC ACC CCA TGG CCC AGC-3′; NM_001815 forward, 5′-CAC CAA TGC ATC CCT GCT GAT CCA-3′ and reverse, 5′-GCT GGT TCT TCC AGT TTT GGC AAG G-3′; NM_015714 forward, 5′-GCG CCG TGC CAC TAA GGT CAT TC-3′ and reverse, 5′-AGA CGT CTG GCG GCC GTG AA-3′; NM_033027 forward, 5′-GCA GTG GAA GCT TTC GGC AGC-3′ and reverse, 5′-AGC TCC CGC TTC TCC TCC CG-3′.

The following conditions were applied for the PCR amplification reactions: an initial incubation step at 95 °C for 2 min, followed by 40 amplification cycles, each one consisting of a denaturation step at 95 °C for 10 s, an annealing step at 60 °C for 10 s, and an extension step at 72 °C for 30 s. Following this reaction, a melting curve analysis was performed in order to verify the specificity of the amplicon. The StepOne™ Real-Time PCR Systems software supplied by the instrument manufacturer (Applied Biosystems) was used for processing and analysis of the data. Diluted aliquots (1/10, 1/100) of the cDNA reaction mixture were analyzed for generation of a standard curve. Relative amount of mRNA was quantified using the freshly prepared standard curve.

### Statistical analyses

Results are expressed as the mean ± standard deviation (SD). All statistical analyses were performed using SPSS software (Version 20, SPSS Inc, Chicago, IL, USA). One-way ANOVA was used for comparison of differences among the three oriental obesity patterns. Independent student’s *t* test was used for comparison of differences between two groups. Correlations between clinical parameters and the level of gene expression were assessed by Pearson’s correlation test. To determine correlation values, heat map analyses were performed using PermutMatrix software (Version 1.0.3 EN, PermutMatrix, Montpellier, France). P value <0.05 was considered statistically significant. As mentioned above, gene ontology (GO) term enrichment was analyzed using tools available on the DAVID website [35] and data from ‘GOTERM_BP_ALL’ database are presented in Table 1.

**Table 1:** Gene ontology analysis of cluster 1

Biological terms	Count	%	P-value	Bonferroni	Benjamini	False discovery rate (FDR)
Defense response	31	18.24	5.17E−13	6.85E−10	6.85E−10	8.46E−10
Inflammatory response	19	11.18	5.27E−09	6.99E−06	3.50E−06	8.63E−06
Response to wounding	22	12.94	9.11E−08	1.21E−04	4.03E−05	1.49E−04
Behavior	20	11.76	2.84E−07	3.76E−04	9.40E−05	4.64E−04
Chemotaxis	12	7.06	7.09E−07	9.40E−04	1.88E−04	0.001161
Taxis	12	7.06	7.09E−07	9.40E−04	1.88E−04	0.001161
Immune response	23	13.53	1.78E−06	0.00236013	3.94E−04	0.002917
Response to bacterium	11	6.47	2.81E−05	0.03663204	0.00531722	0.046055
Locomotor behavior	12	7.06	1.14E−04	0.14035727	0.01872723	0.186507
Female pregnancy	7	4.12	9.08E−04	0.70026536	0.12529893	1.476213
Learning or memory	7	4.12	9.52E−04	0.7172579	0.118669	1.547164
Defense response to bacterium	7	4.12	9.98E−04	0.73386108	0.11338081	1.62068

## Results

### Anthropometric and body composition parameters, and oriental obesity patterns of the subjects

The identification numbers, age, sex, general characteristics, anthropometric and body composition parameters, and oriental obesity patterns of the 23 subjects (19 females and 4 males) involved in the present microarray study are shown in Table 2. Among the subjects, 9 showed symptoms of LDS (3 males and 6 females), 8 exhibited FAS pattern (1 male and 7 females) and 6 showed symptoms of YDS (all were females) (Table 3). Statistical analysis using one-way ANOVA revealed no significant differences in the obesity-related parameters among these three disease patterns (Table 3). However, since there are possibilities that age and sex of the subjects might influence the bias, we further extended our statistical evaluation using ANCOVA being adjusted by these two parameters as covariates. The results reconfirmed no significant differences in the obesity-related parameters (Table 3), consistent with our ANOVA analysis. This assures that in our study, the selection bias was minimized by appropriate randomization. Further, to judge whether the distributions of TG and HDL-C might be skewed, we also carried out the non-parametric method of Kruskal–Wallis test for these two parameters. The analysis (P values: 0.722 and 0.319 for TG and HDL-C, respectively) did not show any difference from our evaluation using parametric method.

**Table 2:** Basic characteristics, anthropometric, and body composition parameters of the subjects

Patient ID	Pattern	Age (years)	Sex	Height (cm)	BW (kg)	BMI (kg/m^2^)	MR (kcal/day)	WC (mm)	FP (%)	TBF (%)	FBS (mg/dL)	TG (mg/dL)	HDL (mg/dL)	T.Chol (mg/dL)	sBP (mmHg)	dBP (mmHg)	PR (/min)
2268	YDS	53	F	163	70.8	26.6	1840	91.5	34.5	24.4	98	74	39	170	140	72	88
0268	YDS	43	F	157	71.1	28.8	1740	92.7	35.3	25.1	110	183	183	206	134	86	68
3409	YDS	54	F	148	64.6	29.5	1230	98.6	43.4	28	94	72	73	194	126	83	70
5170	FAS	31	F	162	76.6	29.2	2020	95	41.9	32.1	92	106	49	187	108	78	75
9854	LDS	26	M	180	99	30.6	1980	104	25.2	28.1	98	176	34	200	129	78	69
0102	LDS	54	M	168.4	80	28.2	1380	101.2	32.1	24.4	92	94	94	288	130	88	68
3510	YDS	50	F	161	70.5	27.2	1030	97.5	38	26.8	101	189	73	276	149	82	78
6196	YDS	36	F	160.7	78.6	30.4	1250	100	39.6	31.1	92	88	55	176	123	82	66
8289	LDS	43	F	160.2	83.2	32.4	2170	98.5	43.1	35.8	111	121	38	181	120	82	68
8523	LDS	47	F	163.4	81.3	30.4	1730	104	36.1	29.3	102	56	59	189	128	79	72
9037	YDS	32	F	164.5	75.6	27.9	1620	94.4	42.6	32.2	94	95	63	193	127	71	89
0023	FAS	41	F	155	64.7	26.9	1370	86.2	36	23.3	88	81	54	204	135	89	69
2998	LDS	26	M	172	81.1	27.4	2180	100.5	31.6	26.5	95	172	79	157	142	78	82
2639	FAS	29	F	160	77.3	30.2	1410	103	49.3	38.1	101	155	56	226	132	81	78
6987	FAS	37	F	163.1	72.9	27.4	1150	89	38.9	28.3	111	99	47	167	118	81	76
7202	FAS	39	F	157.2	67.6	27.4	1030	94.6	38.6	26.1	82	95	57	139	129	78	80
9768	LDS	43	F	157.6	66.5	26.8	1060	84	37.1	24.7	92	88	50	183	115	73	61
9933	LDS	39	F	160	78.9	30.8	1070	103.5	37.7	29.8	96	99	44	163	121	82	68
9990	FAS	44	M	180.7	92.9	28.5	1660	102	26.6	24.8	93	88	44	192	122	76	56
7276	FAS	36	F	170	78.2	27	1890	96	35.5	27.7	86	82	94	199	108	72	68
7887	LDS	48	F	156.1	70.1	28.8	1490	94.7	41.6	29.1	76	118	60	320	133	86	72
9848	FAS	41	F	158	74.9	30	1700	96.8	39	29.2	108	188	39	178	119	83	68
2382	LDS	42	F	158.5	73.9	29.4	1480	106	35.2	26	109	238	31	189	108	74	98

*BW* body weight, *BMI* body mass index, *MR* metabolic rate, *WC* waist circumference, *FP* fat percentage, *TBF* total body fat, *FBS* fasting blood sugar, *TG* triglyceride, *HDL* high density lipoproteins, *T. chol* total cholesterol, *sBP* systolic blood pressure, *dBP* diastolic blood pressure, *PR* pulse rate, *YDS* yang deficiency syndrome, *FAS* food accumulation syndrome, *LDS* liver depression syndrome

**Table 3:** Baseline characteristics of the three most frequent oriental obesity patterns: yang deficiency syndrome (YDS), food accumulation syndrome (FAS), and liver depression syndrome (LDS)

Gender	YDS (n = 6)	LDS (n = 9)	FAS (n = 8)	Total (n = 23)	*P* value	*P* ^2^ value
	Male = 0/Female = 6	Male = 3/Female = 6	Male = 1/Female = 7	Male = 4/Female = 19		
Age (years)	44.7 ± 9.20	40.9 ± 9.47	37.3 ± 5.15	40.6 ± 8.30	0.262	
BW (kg)	71.9 ± 4.81	79.3 ± 9.28	75.6 ± 8.46	76.1 ± 8.28	0.650	0.764
BMI (kg/m^2^)	28.4 ± 1.44	29.4 ± 1.80	28.3 ± 1.34	28.8 ± 1.58	0.670	0.843
MR (kcal/day)	1451.7 ± 325.54	1615.6 ± 428.34	1528.8 ± 348.44	1542.6 ± 365.98	0.635	0.752
WC (mm)	95.8 ± 3.42	99.6 ± 6.77	95.3 ± 5.73	97.1 ± 5.83	0.619	0.484
FP (%)	38.9 ± 3.68	35.5 ± 5.43	38.2 ± 6.38	37.3 ± 5.39	0.809	0.067
TBF (%)	27.9 ± 3.16	28.2 ± 3.48	28.7 ± 4.67	28.3 ± 3.70	0.741	0.051
FBS (mg/dL)	98.2 ± 6.65	96.8 ± 10.38	95.1 ± 10.51	96.6 ± 9.26	0.473	0.848
TG (mg/dL)	116.8 ± 54.29	129.1 ± 56.19	111.8 ± 38.83	119.9 ± 48.57	0.418	0.590
HDL (mg/dL)	81.0 ± 51.57	54.3 ± 21.17	55.0 ± 16.92	61.5 ± 31.60	0.126	0.545
T.Chol (mg/dL)	202.5 ± 38.30	207.8 ± 56.69	186.5 ± 26.08	199.0 ± 42.55	0.197	0.162
sBP (mmHg)	133.2 ± 9.91	125.1 ± 10.20	121.4 ± 10.23	125.9 ± 10.75	0.994	0.707
dBP (mmHg)	79.3 ± 6.25	80.0 ± 5.02	79.8 ± 5.06	79.7 ± 5.13	0.600	0.832
PR (/min)	76.5 ± 10.15	73.1 ± 10.86	71.3 ± 7.72	73.3 ± 9.47	0.711	0.972

Data are shown as Mean ± SD

*P* value: One-way ANOVA was used for cross-sectional comparison among three groups

*P* ^2^ value: P value from ANCOVA adjusted by age and sex

*BW* body weight, *BMI* body mass index, *MR* metabolic rate, *WC* waist circumference, *FP* fat percentage, *TBF* total body fat, *FBS* fasting blood sugar, *TG* triglyceride, *HDL* high density lipoproteins, *T. chol* total cholesterol, *sBP* systolic blood pressure, *dBP* diastolic blood pressure, *PR* pulse rate

### Validation of specific syndrome differentiation by gene clustering of the obese patients

**Fig. 1:**
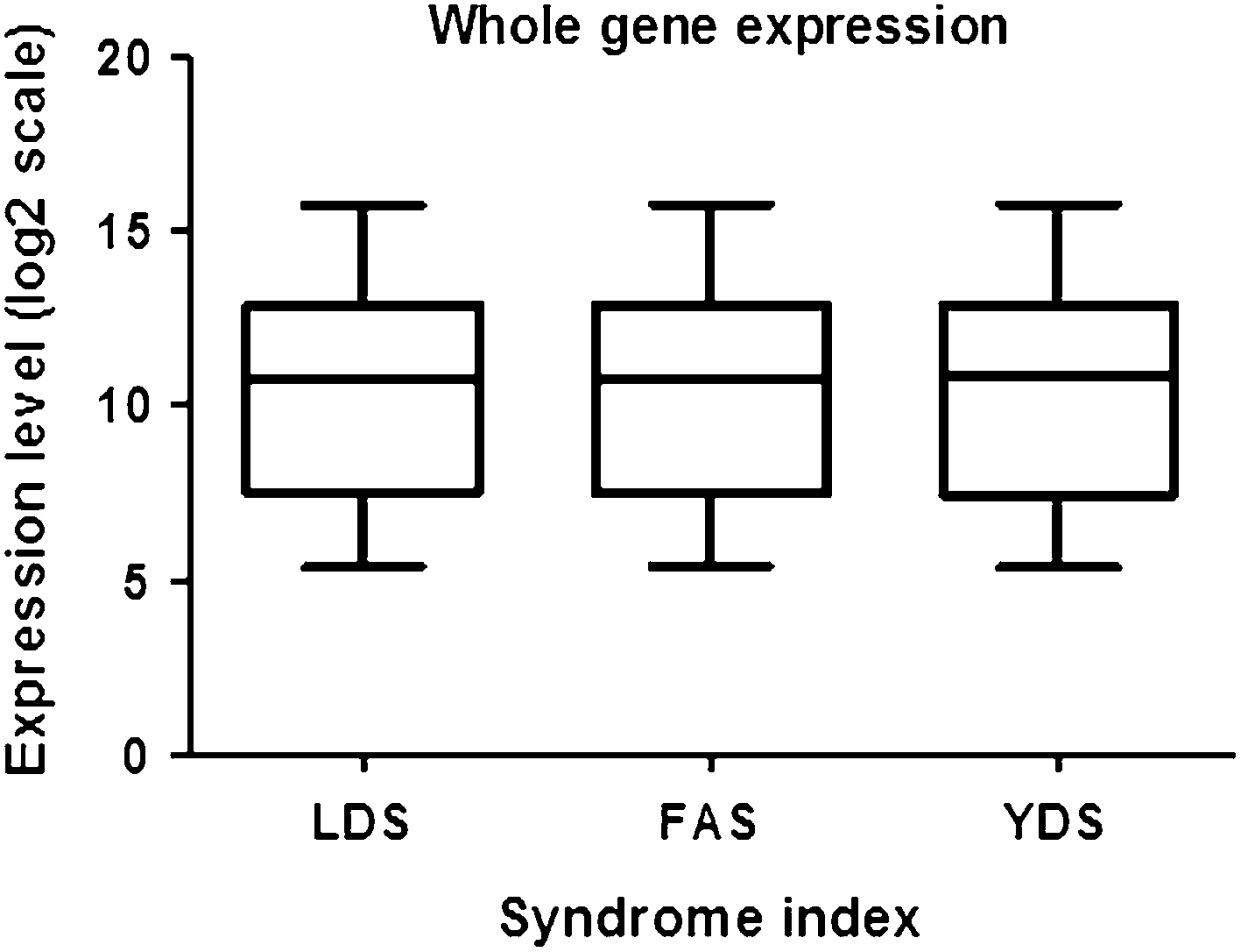
Whole gene expression of subjects representing three distinct oriental obesity patterns (LDS, FAS, and YDS). The average gene expression profile from nine subjects of LDS, eight of FAS and six of YDS patterns are represented by *box plot* (each showing 5th, 25th 50th, 75th, and 95th percentiles)

**Fig. 2:**
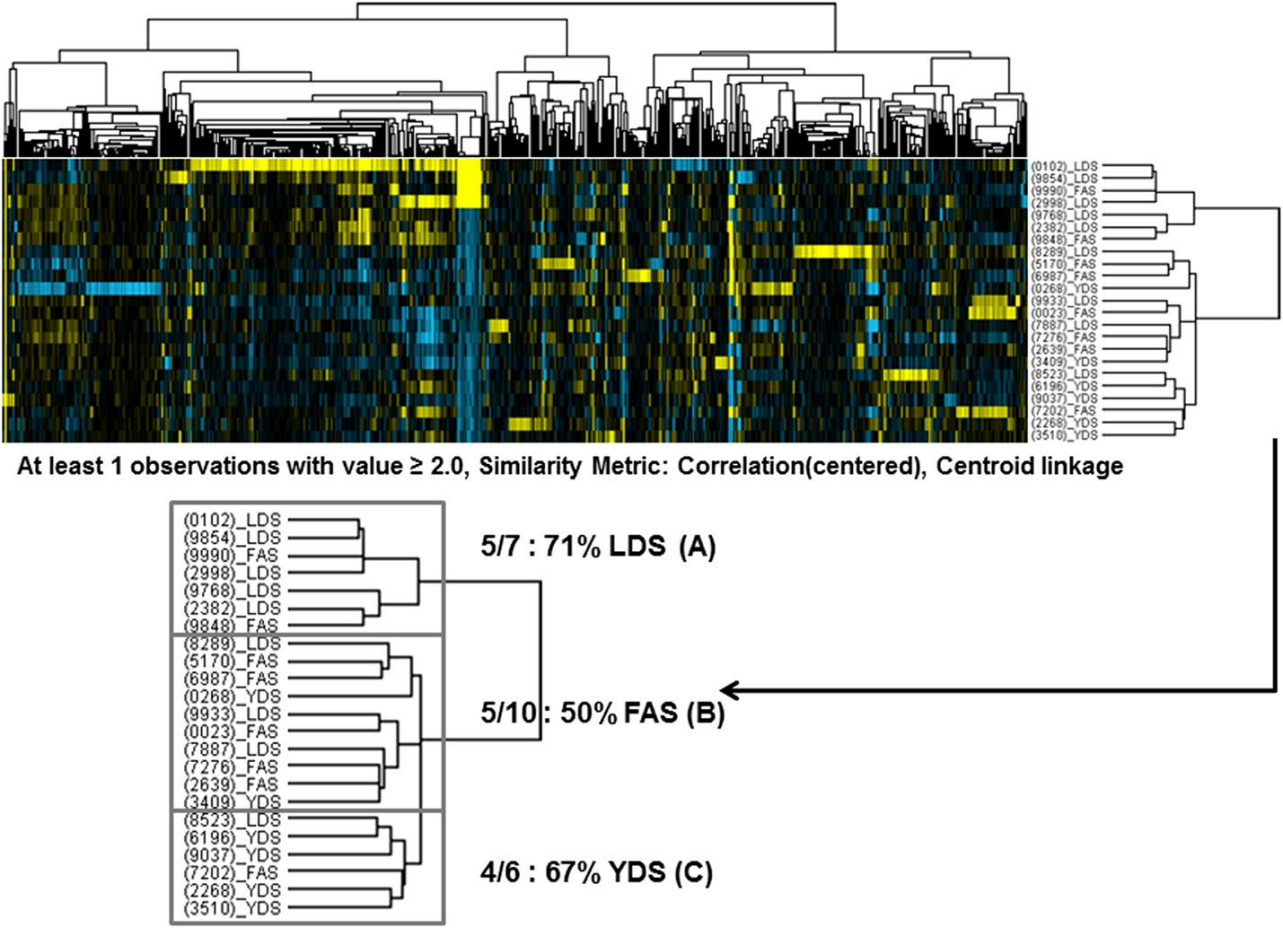
Classification of three distinct oriental obesity patterns based on gene clustering analysis. Whole genes and arrays (three oriental obesity patterns: LDS, FAS, and YDS) were filtered and clustered following a specific condition (at least one observation with value >−2.0), similarity metric [Correlation (centered)] and clustering method (centroid linkage). Clusters of genes or arrays are represented by Treeview

DNA microarray analysis of the PMBC of the subjects showed no marked difference in the average expression of the probes on the array among the three syndrome indexes mentioned above (Fig. 1), indicating that the quality of the performed microarray assay was satisfactory. When we performed the gene clustering analysis, those three oriental obesity patterns were categorized into three entities (Fig. 2). We explored various filtering conditions for clustering analysis. In most of these tested filtering conditions, clustering of genes in a syndrome index-dependent manner was found. Here we present the clustering results based on one of the filtering conditions as described above where we observed optimum syndrome index dependency. More specifically, three marked classifications were evident when whole genes were filtered according to a specific condition (at least one observation with value ≥2.0), similarity metric [correlation (centered)] and clustering method (centroid linkage). The categories were further divided into two parts. One part was comprised of seven subjects among whom five (71 %) represented LDS pattern (A) and two (29 %) belonged to the FAS pattern type (Fig. 2). The other part representing the remaining 16 subjects was further divided into two groups. One group consisted of 10 subjects among whom five represented the FAS pattern (50 %, B) and the other group consisted of six subjects among whom four (67 %) exhibited the YDS pattern (C).

### Identification of differentially expressed genes (DEGs) in specific oriental obesity pattern

**Fig. 3:**
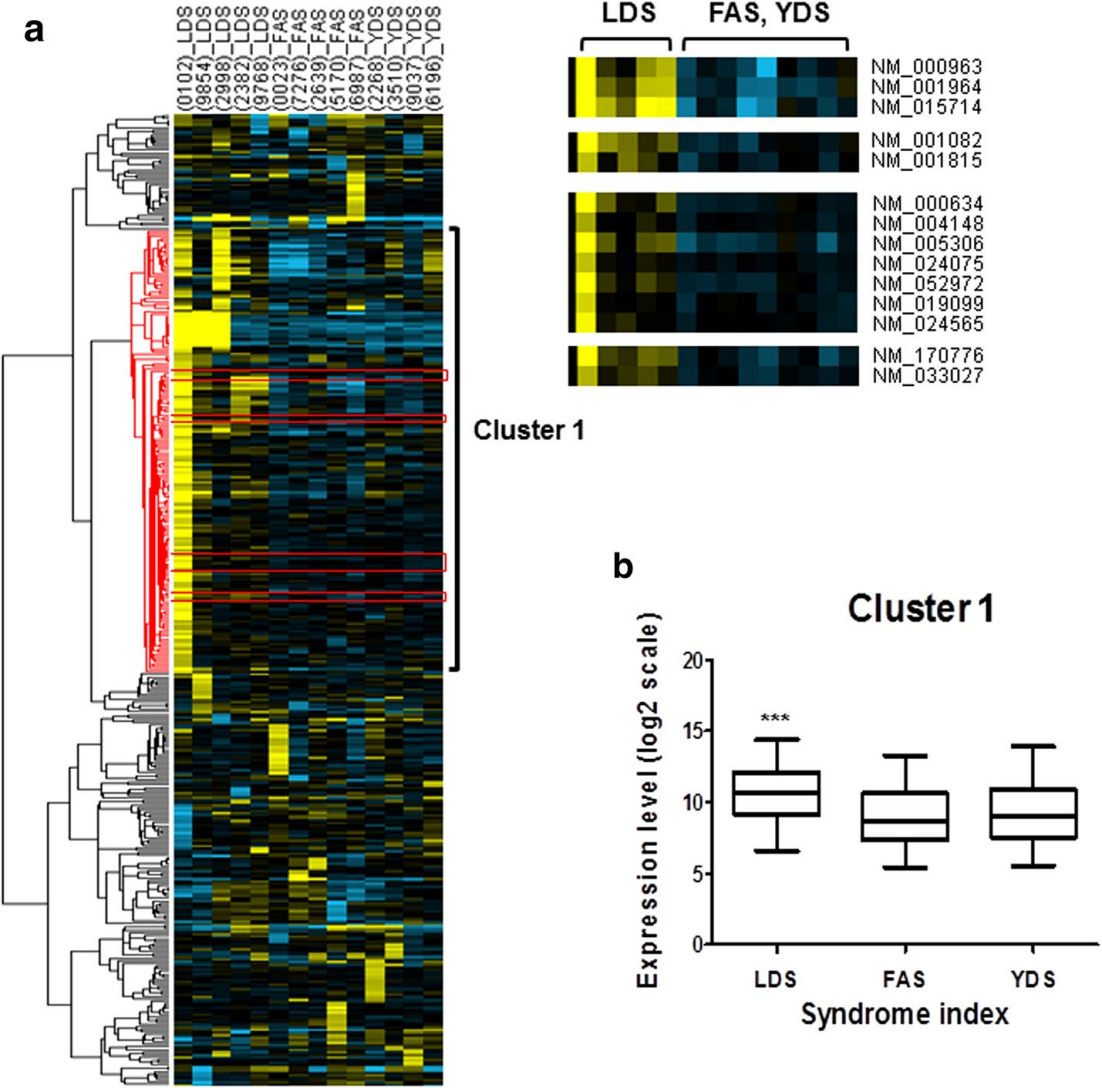
Identification of oriental obesity pattern specific genes. **a** 382 filtered genes of five subjects of LDS, five subjects of FAS, and four subjects of YDS patterns were clustered and represented by Treeview following a specific condition (at least one observation with value ≥2.0), similarity metric [correlation (centered)] and clustering method (centroid linkage). Cluster 1 represents classified genes specifically upregulated in LDS. A total of 14 genes that were commonly upregulated in LDS pattern were sorted out from cluster 1 (*right panel*). **b** Expression of cluster 1 genes is represented by box plot (each showing 15th, 25th, 50th, 75th, and 95th percentiles). P-value was determined using one-way ANOVA analysis (***P < 0.0001)

For identification of gene sets that are presumed to be differentially regulated among the classified oriental obesity patterns, we performed further gene clustering analysis using the microarray data. As shown in Fig. 2, significant gene sets for each pattern were not detected when all gene expression profiles of 23 subjects were processed for clustering analysis. This result suggests that each pattern may consist of subtype patterns which differ from each other in their gene expression profiles. In this regard, we narrow down numbers of expression profiles based upon clustering results in Fig. 2. More specifically, we performed gene clustering analysis using the microarray data of commonly upregulated genes from the categorized five subjects of LDS (A), five subjects of FAS (B), and four subjects of YDS patterns (C), as mentioned above (Fig. 2). A total of 382 genes were subjected to clustering analysis (Fig. 3a, left panel, the list of genes is depicted in Additional file 2: Table S2) which ultimately revealed that among these genes, 176 were specifically clustered (cluster 1).

**Fig. 4:**
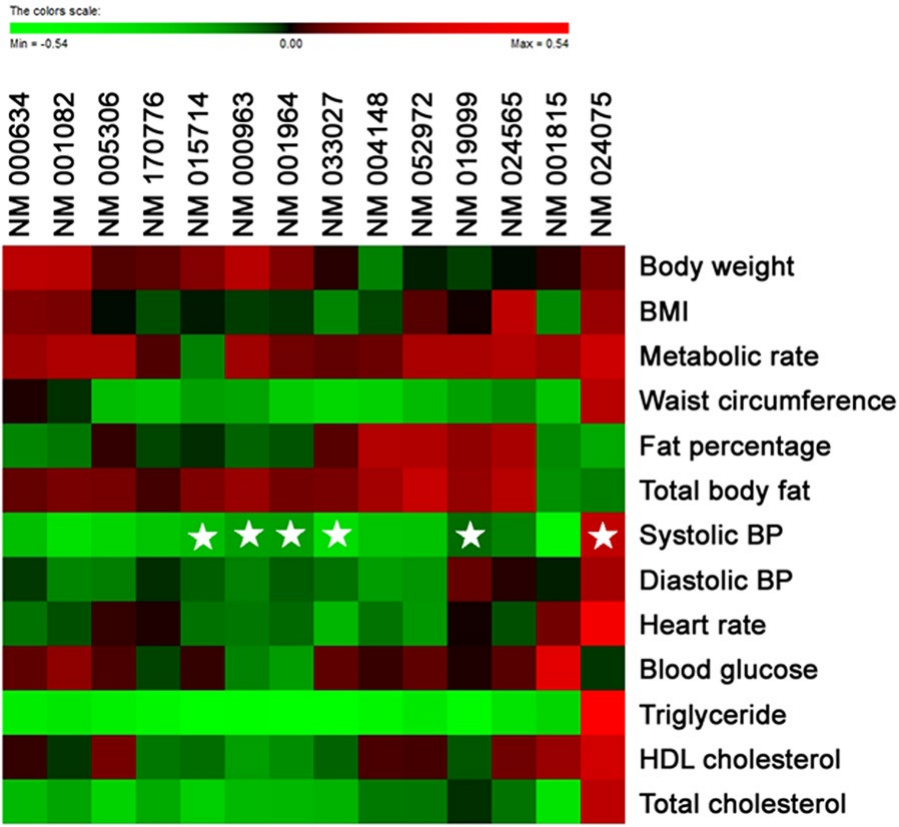
Analyses of correlation between the selected genes and obesity parameters using a heat map. As the color scale is shown, *green color* indicates a negative correlation, while *red color* indicates a positive correlation (a symbol of *asterisk* indicates statistical significance of P < 0.05)

**Fig. 5:**
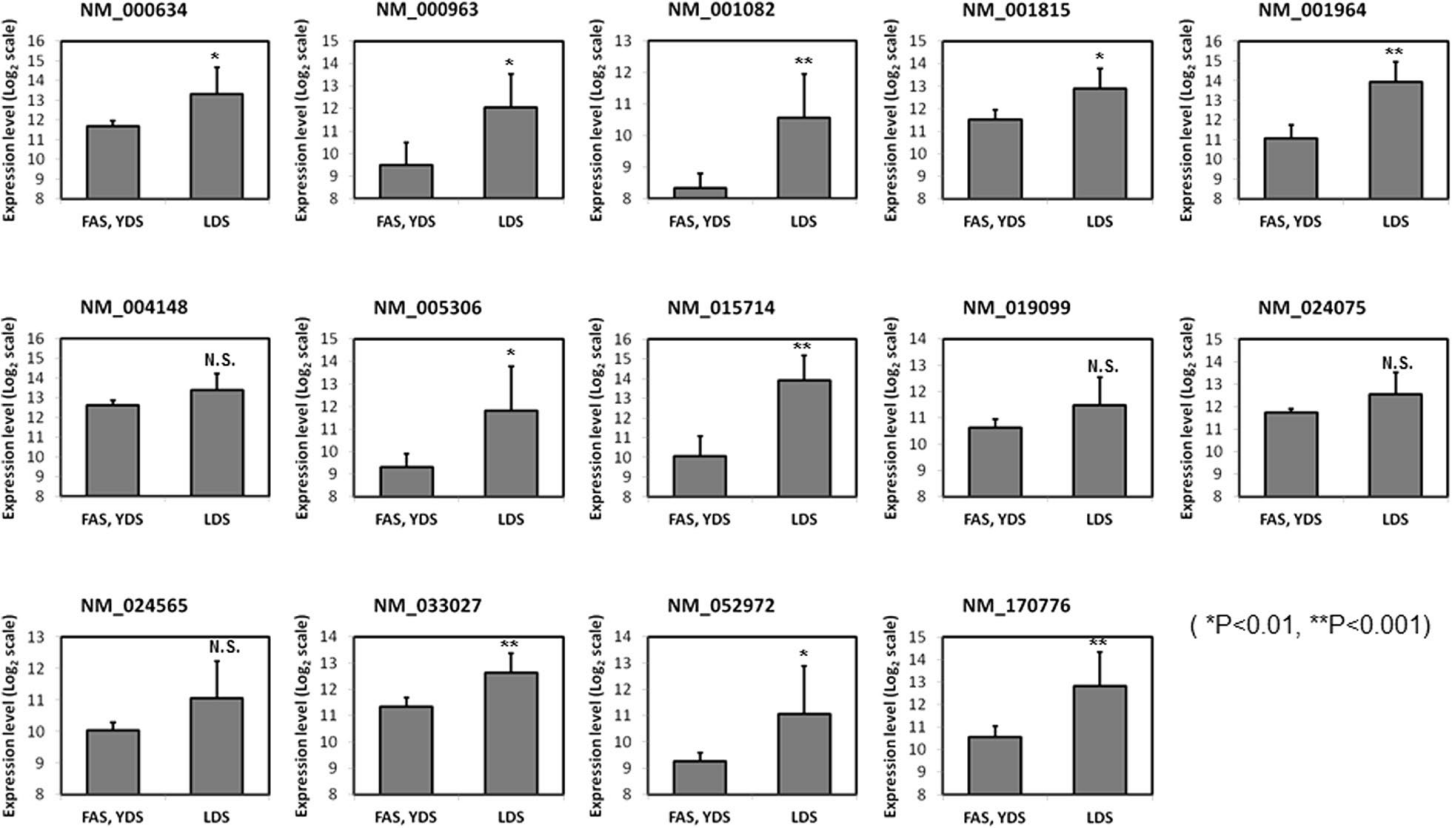
Expression of 14 selected genes in 14 subjects represented by LDS (five subjects), FAS (five subjects), and YDS (four subjects) patterns. The selected 14 genes were sorted out from each individual DNA microarray data of the subjects and their expression level was compared between subjects of LDS and FAS and YDS in combination. P-values were determined using student’s t-test (*N.S.* not significant; *P < 0.01, **P < 0.001)

Our analysis also demonstrated that cluster 1 was significantly upregulated (P < 0.0001) only in subjects representing LDS pattern compared to volunteers belonging to FAS and YDS pattern types (Fig. 3a, left panel, Fig. 3b). According to gene ontology analysis, genes that were highly enriched in this cluster were found to be related to defense response against pathogens or chemical insults (Table 1). Next, from all of the genes grouped in cluster 1, we sorted a total of 14 genes that were commonly up-regulated in LDS subjects (Fig. 3a, right panel). The identification number and name of these genes are shown in Table 4. Correlation between these 14 genes and the obesity parameters of the aforementioned 14 subjects as analyzed by heatmap plots is shown in Fig. 4. To determine whether the increase in expression of these 14 genes in LDS pattern was significant, we sorted them from the microarray data set of those 14 individuals and performed statistical analysis using student’s t-test. Accordingly, our data showed that among those 14 selected genes, the expression of 10 genes was significantly higher in LDS obesity pattern compared to FAS and YDS patterns (Fig. 5).

**Table 4:** Gene ID and description of 14 sorted genes

Gene ID	Gene name
NM_170776	G protein-coupled receptor 97 (GPR97)
NM_015714	G0/G1 switch 2 (G0S2)
NM_001815	Carcinoembryonic antigen-related cell adhesion molecule 3 (CEACAM3)
NM_019099	Chromosome 1 open reading frame 183 (FAM212B)
NM_024565	Cyclin J-like (CCNJL)
NM_033027	Cystein-serine-rich nuclear protein 1 (CSRNP1)
NM_001082	Cytochrome P450, family 4, subfamily F, polypeptide 2 (CYP4F2)
NM_001964	Early growth response 1 (EGR1)
NM_005306	Free fatty acid receptor 2 (FFAR2)
NM_000634	Interleukin 8 receptor alpha (CXCR1)
NM_052972	Leucine-rich alpha-2-glycoprotein 1 (LRG1)
NM_004148	Ninjurin 1 (NINJ1)
NM_000963	Prostaglandin-endoperoxide synthase 2 (prostaglandin G/H synthase and cyclooxygenase, PTGS2)
NM_024075	tRNA splicing endonuclease 34 homolog *Saccharomyces cerevisiae* (TSEN34)

### Confirmation of higher expression of particular genes in specific oriental obesity pattern

**Fig. 6:**
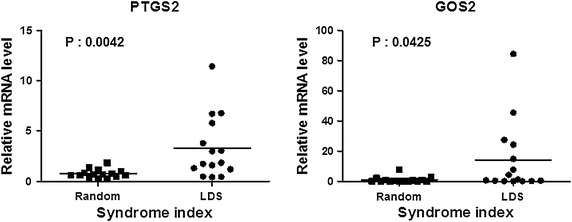
Expression levels of endogenous PTGS2 and G0S2 genes in 15 randomly selected subjects (Random) or 15 volunteers representing LDS pattern (LDS) were measured using quantitative RT-PCR. The data are represented by *scatter plot*. P-value was determined using student’s t-test

Next, we made an effort to further validate the higher expression of the identified 10 marker genes mentioned above in LDS obese patients (Fig. 5). For this, we selected five genes (NM_000963, prostaglandin endoperoxide synthase 2, PTGS2; NM_015714, G0/G1 switch 2, G0S2; NM_001815, carcinoembryonic antigen-related cell adhesion molecule 3, CEACAM3; NM_033027, cystein-serine-rich nuclear protein 1, CSRNP1; and NM_000634, interleukin 8 receptor, alpha, CXCR1) as representatives and measured their expression individually by quantitative RT-PCR using PBMC samples of the subjects. In this experiment, 15 LDS PBMC samples and 15 randomly selected PBMC samples (for PTGS2 and G0S2), and 10 LDS PBMC samples and 10 randomly selected PBMC samples (for CEACAM3, CSRNP1, and CXCR1) collected from the additional subjects were used for the gene expression profiling. The age, gender, and the clinical features of these subjects are depicted in Additional file 3: Table S3 and Additional file 4: Table S4, respectively. As shown in Fig. 6 and Additional file 5: Figure S1, significantly higher expression of all of the above mentioned five genes was observed in the LDS obese patients compared to the randomly selected obese subjects. Further to examine whether the distribution of the relative mRNA level of PTGS2 might be skewed, we carried out the statistical analysis of this parameter using non-parametric method of Mann–Whitney’s U test. The analysis further confirmed significantly higher PTGS2 expression in LDS compared to random samples (P = 0.03). The relative expression of PTGS2 and G0S2 genes in the 15 LDS and 15 randomly selected subjects are depicted individually in Additional file 6: Table S5 and Additional file 7: Table S6, respectively.

## Discussion

Obesity, which was officially considered a disease by the World Health Organization (WHO) in 1997 [37], is a serious and alarming global public health problem. Accumulating evidence suggests that obesity can increase the risk of many other life-threatening diseases, including certain types of cancers. WHO predicts that approximately 2.3 billion adults in the global population will be overweight by 2015 and 700 million of them will be obese [38]. Notably, obesity is also becoming a major concern in South Korea as The Korea National Health and Nutrition Examination Survey reported an overall incidence of Korean adult obesity of 30.7 % in 2008, compared with 21.8 % in 1998 [14].

In the entire human population, each individual has his or her own entity, which differs from that of others. The fundamental concept in the practice of oriental medicine is based on the human constitution, which is classified according to the physical, physiological, and clinical characteristics of the individual. According to this concept, individuals from different constitutional patterns differ in their metabolic pattern and susceptibility to disease with a varying degree. As a norm, clinicians in oriental medicine focus on evaluation of the cause, nature, and location of the disease according to the signs and symptoms shown by the patients [12], and, based on this information prescribe individualized treatment to the patients [15]. Interestingly, emerging scientific evidence indicates a genetic basis for oriental medicine, where it has been opined that further investigations using up to date genomic tools could help to introduce oriental medicine as an alternative to personalized treatment practiced in western medicine.

In general, oriental obesity patterns are clinically judged by the oriental medical doctors on the basis of the patient’s current symptoms, personality, digestive or circulatory conditions. According to the Korea Institute of Oriental Medicine, among the Korean population, there are six disease patterns in obese people [26], as follows: SDS, PFS, YDS, FAS, LDS, and BSS. Although the pattern identification is regarded as rather empirical from the scientific point of view, the questionnaire for obesity pattern identification has been validated in various studies [26, 28, 31, 32]. In general, SDS represents a condition for deficiency of the spleen, such as spleen qi deficiency, spleen yang deficiency, and spleen yin deficiency [15]. PFS depicts (1) a condition for retention of phlegm and fluid in any part of the body; (2) a particular state for retention of fluid in the gastrointestinal tract, e.g., gastric retention in pyloric stenosis (WHO). YDS represents a pathological condition characterized by deficiency of the body’s yang qi, which leads to abatement of functions, decline in metabolic activity, and decreased body reactions as well as deficiency-cold manifestations [15]. FAS denotes a pattern/syndrome marked by epigastric and abdominal distention, vomiting of sour matter, anorexia, offensive odor of stools, and curdy and slimy tongue coating [15]. LDS represents a state of stagnation of qi in the liver caused by impairment of free coursing [15]. BSS denotes a morbid condition of blood stagnancy in a particular area of the body resulting from sluggish flow of qi, deficiency of qi or blood, trauma, or yin-cold [15].

Inter-individual genetic differences in the human population, which are bestowed by a number of factors, such as single nucleotide polymorphisms [39], copy number variations [40], and epigenetic or gene expression modifications [41–43], play a vital role in the contribution to phenotypic variations. These variations influence an individual’s anthropometric parameters as well as susceptibility to disease and response to the environment [44]. Therefore, in-depth study of genetic variation may facilitate understanding of the mechanisms underlying differential risk of disease and response to drugs in the human population. Indeed, rapid progress in ‘omics’ sciences has opened a new horizon in traditional medicine research. Accordingly, considerable interest has been paid worldwide, including Korea, India, China, and Japan to studies for understanding the genomic, proteomic, and metabolomic basis of disease patterns.

In recent years, DNA microarray has emerged as a popular technique for investigation of human diseases since it provides unique data about disease mechanisms, regulatory pathways, and gene functions [44]. Microarrays have been employed for investigation of several pathological conditions, such as inflammation, atherosclerosis, breast cancer, colon cancer, and pulmonary fibrosis [45]. In parallel, microarray-based techniques have also been applied in earlier studies to characterize the mode of gene expression in traditional medicinal constitutions and disease patterns. An earlier study attempted to address a scientific approach for oriental medicine treatment by combining oriental therapy for obesity, such as oral administration of *Ephedra sinica* extract or *Sinomenium acutum* extract, and western techniques including DNA microarray [46]. However, the empirical and inherent properties of oriental medicine are not well understood, which prompted us to conduct the current study, where we performed DNA microarray analysis using PBMC samples from obese patients in order to categorize specific symptoms in accordance with oriental obesity patterns.

In this study, we acquired genome-wide transcription profiles of 23 subjects who represented the three most frequent oriental obesity pattern types: FAS, LDS, and YDS. According to our microarray data, no marked difference in the average expression of the probes on the array was evident among the three obesity patterns mentioned above (Fig. 1), indicating that the quality of the performed microarray assay was high. When the all genes and array data were filtered and clustered following a specific condition, the subjects were agglomerated into three main categories represented by LDS, FAS, and YDS patterns, respectively. Thus, our results may be considered as an evidence-based approach addressing a possible molecular explanation for obesity pattern differentiation which is normally practiced in oriental medicine.

A number of recent studies have been conducted to understand the possible genetic explanation for the differentiated constitution types or disease patterns in traditional medicine. These include a microarray survey which identified 785 up-regulated genes and 954 down-regulated genes in YDS in comparison with normal constitution [29]. In another study, analysis of genome wide expression through cDNA microarrays revealed a number of differentially expressed genes in each category of individuals from the three most contrasting constitution types (Vata, Pitta, and Kapha) as defined by Ayurveda-based method in India [30]. On the other hand, genes such as angiotensin I converting enzyme, parathyroid hormone, tumor necrosis factor, CD4, glucose phosphate isomerase, C-reactive protein, and vascular endothelial growth factor have been found to show strong relationships with Kidney-Yang Deficiency syndrome [47].

We further performed gene clustering analysis using the microarray data of commonly categorized five subjects representing LDS, five subjects exhibiting FAS, and four subjects of the YDS. As a result, 176 genes were clustered (cluster 1) and showed significant up-regulation (P < 0.0001) in subjects representing LDS pattern compared to subjects belonging to FAS and YDS pattern types, suggesting that stagnation of qi in the liver may contribute to the etiology of obesity. Indeed, liver stagnation, which is generally caused by prolonged strong emotions or depression, leads to disharmony between the spleen and liver, ultimately giving rise to fluid retention [48]. Because liver becomes depressed in this condition, the gall bladder is also depressed and exhausted. As a result, the ebb and flow of these organs become unbalanced, and the qi mechanism does not flow freely. As a consequence, transformation of fat turbidity becomes difficult and over the course of time the condition leads to obesity [48].

In an earlier study, 12 proteins were differentially expressed in the serum of patients with liver stagnation syndrome, compared with the normal control group [49]. Further analysis revealed that these differential proteins were mainly related to immunization, neuroendocrine and nutrient metabolism. While, in our study, gene ontology analysis of microarray data demonstrated that in cluster 1, where all 176 genes were significantly upregulated in LDS, the genes related to defense response against pathogens or chemical insults were highly enriched, suggesting a possible functional role of these genes in response to the onset and development of liver stagnation. As liver is involved in neutralization and detoxification of harmful chemicals or pathogens, the up-regulation of defense-system related genes in LDS is not an unexpected phenomenon.

For more extensive analysis of our microarray data, we sorted 14 genes from the aforementioned cluster 1 that were commonly up-regulated in LDS. Results showed that expression of 10 of those 14 genes was significantly elevated in LDS compared to FAS and YDS patterns. Correlation between these 14 genes and the obesity parameters of the aforementioned 14 subjects as analyzed by heatmap plots is shown in Fig. 4. Most notable finding in correlation analysis was that among 14 up-regulated genes in LDS subjects, 6 genes showed significant correlation with systolic blood pressure and most of the genes except NM024075 (TSEN34) displayed a tendency of negative correlation. It is widely known in oriental medicine that LDS, or liver qi stagnation in other word, is related to chronic restraint of stress [50, 51] which ultimately leads to liver disease and stress related symptoms including high blood pressure. Although these genes have not yet found to have a direct effect on blood pressure, this may provide a reasonable evidence for oriental medical theory on which further study will be needed.

Our further study revealed that the expression of five representative members of these 10 genes (NM_000963, PTGS2; NM_015714, G0S2; NM_001815, CEACAM3; NM_033027, CSRNP1; and NM_000634, CXCR1) as determined by quantitative RT-PCR was significantly higher in LDS obese patients compared to randomly selected samples. Notably, a number of studies have reported the impact of PTGS2 and G0S2 genes on hepatic function and pathophysiology. The cyclooxygenases 2-prostanoid pathway has been reported to play vital and complex roles in pathogenesis of various liver diseases [52]. A number of studies have indicated that the COX2-prostanoid pathway may inhibit hepatic fibrogenesis by decreasing proliferation, migration, and contractility of hepatic stellate cells [52]. On the other hand, recent studies have reported that targeted disruption of G0S2 alters hepatic energy balance [53] and overexpression of this gene induces triglyceride accumulation in the mouse liver leading to development of hepatic steatosis [54]. While emerging evidence indicates possible linkage between CXCR1, CEACAM3, and CSRNP1 genes and liver function. CXCR1 represents the cellular targets of IL-8 [55] which is known to be involved in the pathogenesis of chronic liver disease. Furthermore, it has been reported that CXCR1 expression is induced in hepatocytes after ischemia/reperfusion (I/R) injury [56]. The results suggests that CXCR1 facilitates repair and regenerative responses of liver after I/R injury. It has been found that CXCR1/CXCR2 ligands promote neutrophil migration to the liver and regulate hepatocyte survival and proliferation [57]. CEACAM3 belongs to CEACAMs which are a group of mammalian immunoglobulin related glycoproteins. They play an important role in cell–cell recognition and modulation of cellular processes such as the shaping of tissue architecture and neovascularization, as well as regulation of hepatic insulin sensitivity by facilitating insulin metabolism in liver [58, 59]. Accumulating evidence indicates that CEACAM3 is critically involved in the opsonin-independent recognition and elimination of several Human-specific bacterial pathogens [60, 61]. Considering the above findings on LDS together, our study can be considered as a probable molecular explanation of pattern-specific pathophysiological condition categorized in oriental obesity medication.

Our study has some limitations. First, although our study can be considered as exploratory, the number of subjects in the present microarray-based study is not large. Data based on larger samples are scientifically more valid compared to smaller samples (being provided that all other conditions are equal) because larger samples minimize the probability of errors and maximize the accuracy and precision of the measurements. Second, we cannot consider that the procedure adopted in this study for pattern identification is absolutely concrete as until now there is no gold standard for the pattern identification in oriental medicine. Third, the obesity patterns identified in this study cannot be explained from the western medicinal point of view as the principle of oriental medicine and conventional medicine are quite different. Fourth, there was a time lag between completion of the study and writing, submission and peer review of the manuscript, all of which can be considerable.

## Conclusion

In conclusion, our study can be considered as an evidence-based approach providing a possible molecular explanation for obesity pattern differentiation practiced in oriental medicine. More specifically, our findings address a novel possibility that the genomic and oriental empirical approaches can be combined and implemented in systematic and synergistic development of personalized medicine. In our study, gene clustering analysis of the microarray data of obese subjects exhibited disease pattern-specific upregulation of expression of several genes in a particular cluster. Even though we were able to identify five marker genes that were specifically expressed in PBMC of patients from LDS obesity pattern, further studies will allow to find more clear evidences that can segregate other oriental obesity patterns. Thus, our study can contribute to the understanding and development of personalized medicine based on oriental obesity pattern.

## Additional files

10.1186/s12967-015-0692-9 Obesity pattern diagnosis questionnaire. The questionnaire consisting of 52 questions addressing systemic symptoms, personality and emotion, digestive function, and circulatory function of the subjects are enlisted.
10.1186/s12967-015-0692-9 List of commonly upregulated genes that were subjected to cluster analysis.
10.1186/s12967-015-0692-9 Clinical characteristics of 15 randomly selected subjects and 15 volunteers representing liver depression syndrome (LDS) pattern the samples of whom were used for the quantitative real-time PCR analyses of prostaglandin endoperoxide synthase 2 (PTGS2), G0/G1 switch 2 (G0S2) genes.
10.1186/s12967-015-0692-9 Clinical characteristics of 10 randomly selected subjects and 10 volunteers representing liver depression syndrome (LDS) pattern the samples of whom were used for the quantitative real-time PCR analyses of carcinoembryonic antigen-related cell adhesion molecule 3 (CEACAM3), cystein-serine-rich nuclear protein 1 (CSRNP1), and interleukin 8 receptor, alpha (CXCR1) genes.
10.1186/s12967-015-0692-9 Expression levels of CEACAM3, CSRNP1, and CXCR1 genes in 10 randomly selected subjects (Random) or 10 volunteers representing LDS pattern (LDS) were measured using quantitative RT-PCR. P-value was determined using student’s t-test.
10.1186/s12967-015-0692-9 Expression levels of endogenous PTGS2 gene in 15 randomly selected subjects (Random) or 15 volunteers representing LDS pattern (LDS) as measured by quantitative RT-PCR.
10.1186/s12967-015-0692-9 Expression levels of endogenous G0S2 gene in 15 randomly selected subjects (Random) or 15 volunteers representing LDS pattern (LDS) as measured by quantitative RT-PCR.
