# Supplementary material for: Understanding the molecular aspects of oriental obesity pattern differentiation using DNA microarray
Source: J Transl Med. 2015 Oct 19;13:331. doi: 10.1186/s12967-015-0692-9 (PMC4617455; doi:10.1186/s12967-015-0692-9)
Supplement: Supplementary file 1 — 10.1186/s12967-015-0692-9 Obesity pattern diagnosis questionnaire. The questionnaire consisting of 52 questions addressing systemic symptoms, personality and emotion, digestive function, and circulatory function of the subjects are enlisted. [file 12967_2015_692_MOESM1_ESM.docx]

**Obesity pattern diagnosis questionnaire**

Serial no.

| Date |  | Gender |  | Age |  | Height |  |
| --- | --- | --- | --- | --- | --- | --- | --- |
| Weight |  | BMI |  | Waist circumference |  | Hip circumference |  |

| **General symptoms** | ⑤ very intense | ④  intense | ③ moderate | ② mild | ①little |
| --- | --- | --- | --- | --- | --- |
| 1. I am tired and weak. |  |  |  |  |  |
| 1. I don’t like to move around. |  |  |  |  |  |
| 1. I feel dizzy. |  |  |  |  |  |
| 1. My thinking is not clear and I am confused. |  |  |  |  |  |
| 1. My face is pale. |  |  |  |  |  |
| 1. I have a cold sweat |  |  |  |  |  |
| 1. I am intolerant to coldness. |  |  |  |  |  |
| 1. My hands or feet are cold. |  |  |  |  |  |

| **Personality and emotion** | ⑤ very intense | ④  intense | ③ moderate | ② mild | ①little |
| --- | --- | --- | --- | --- | --- |
| 1. I am introverted. |  |  |  |  |  |
| 1. I am prone to be angry. |  |  |  |  |  |
| 1. I tend to worry. |  |  |  |  |  |
| 1. I feel chest tightness when stressed. |  |  |  |  |  |
| 1. I am depressed. |  |  |  |  |  |
| 1. I feel more pain when stressed. |  |  |  |  |  |
| 1. There is tenderness in my mid-sternum area. |  |  |  |  |  |
| 1. There is a sense of fullness or pain in my flank. |  |  |  |  |  |

| **Digestive function** | ⑤ very intense | ④  intense | ③ moderate | ② mild | ①little |
| --- | --- | --- | --- | --- | --- |
| 1. My digestive function is poor. |  |  |  |  |  |
| 1. I have indigestion that is aggravated due to mental stress. |  |  |  |  |  |
| 1. I frequently eat excessive. |  |  |  |  |  |
| 1. I can’t stop eating even when I am full. |  |  |  |  |  |
| 1. I lost appetite. |  |  |  |  |  |
| 1. I eat less. |  |  |  |  |  |
| 1. I feel bloated. |  |  |  |  |  |
| 1. I use to belch a lot. |  |  |  |  |  |
| 1. I have excessive gas in my abdomen. |  |  |  |  |  |
| 1. I feel food is stuck in esophagus or stomach. |  |  |  |  |  |
| 1. Food remains undigested in my stomach often. |  |  |  |  |  |
| 1. I frequently feel abdominal pain after eating meal. |  |  |  |  |  |
| 1. I often feel nauseated and like to vomit after eating. |  |  |  |  |  |
| 1. I have loose stools. |  |  |  |  |  |
| 1. I suffer from frequent diarrhea. |  |  |  |  |  |
| 1. I usually have water sound in the stomach. |  |  |  |  |  |
| 1. I suffer from constipation. |  |  |  |  |  |
| 1. I have difficulty urinating. |  |  |  |  |  |

| **Circulatory function** | ⑤ very intense | ④  intense | ③ moderate | ② mild | ①little |
| --- | --- | --- | --- | --- | --- |
| 1. My body swells frequently. |  |  |  |  |  |
| 1. I have often swellings in my face or eyelid. |  |  |  |  |  |
| 1. I have often swelling in hands or feet. |  |  |  |  |  |
| 1. I have more swelling in lower-body. |  |  |  |  |  |
| 1. I feel a sharp pain somewhere in my body. |  |  |  |  |  |
| 1. I have persistent pinpoint pain. |  |  |  |  |  |
| 1. I have pain that is aggravated at night. |  |  |  |  |  |
| 1. I have experienced a trauma recently. |  |  |  |  |  |
| 1. I have a bleeding tendency. |  |  |  |  |  |
| 1. I have always bruised easily. |  |  |  |  |  |
| 1. There are dark puffy circles under my eyes. |  |  |  |  |  |
| 1. My skin is scaly. |  |  |  |  |  |
| 1. I have a palpable mass in abdomen. |  |  |  |  |  |
| 1. I have fixed masses somewhere in my body. |  |  |  |  |  |
| 1. I have dysmenorrhea. |  |  |  |  |  |
| 1. I have cough with sputum. |  |  |  |  |  |
| 1. I have sensation of a foreign body in my throat. |  |  |  |  |  |
| 1. I have chest discomfort. |  |  |  |  |  |
