# Supplementary material for: Understanding the molecular aspects of oriental obesity pattern differentiation using DNA microarray
Source: J Transl Med. 2015 Oct 19;13:331. doi: 10.1186/s12967-015-0692-9 (PMC4617455; doi:10.1186/s12967-015-0692-9)
Supplement: Supplementary file 3 — 10.1186/s12967-015-0692-9 Clinical characteristics of 15 randomly selected subjects and 15 volunteers representing liver depression syndrome (LDS) pattern the samples of whom were used for the quantitative real-time PCR analyses of prostaglandin endoperoxide synthase 2 (PTGS2), G0/G1 switch 2 (G0S2) genes. [file 12967_2015_692_MOESM3_ESM.docx]

**Supplementary Table 3. Clinical characteristics of 15 randomly selected subjects and 15 volunteers representing liver depression syndrome (LDS) pattern the samples of whom were used for the quantitative real-time PCR analyses of prostaglandin endoperoxide synthase 2 (PTGS2) and G0/G1 switch 2 (G0S2) genes.**

|  | Random (n=15) | LDS (n=15) | *P* value | *P*^2^ value |
| --- | --- | --- | --- | --- |
| Gender | Male=3, Female=12 | Male=2, Female=13 |  |  |
| Age (yrs) | 38.1 ± 10.20 | 46.7 ± 7.08 | 0.012 |  |
| Height (cm) | 162.9 ± 7.06 | 157.3 ± 6.54 | 0.032 | 0.001 |
| BW (kg) | 75.2 ± 9.18 | 70.1 ± 8.99 | 0.132 | 0.368 |
| BMI (kg/m^2^) | 28.3 ± 2.41 | 28.2 ± 2.24 | 0.950 | 0.481 |
| MR (kcal/day) | 1458.7 ± 303.41 | 1396.7 ± 391.89 | 0.632 | 0.961 |
| WC (mm) | 98.5 ± 9.36 | 96.1 ± 4.69 | 0.377 | 0.338 |
| FP (%) | 36.7 ± 6.34 | 37.3 ± 5.40 | 0.577 | 0.022 |
| TBF (%) | 27.6 ± 6.51 | 26.5 ± 4.45 | 0.567 | 0.118 |
| FBS (mg/dL) | 97.3 ± 7.95 | 98.3 ± 6.55 | 0.710 | 0.995 |
| TG (mg/dL) | 125.2 ± 75.10 | 118.5 ± 49.54 | 0.776 | 0.542 |
| HDL (mg/dL) | 54.3 ± 15.69 | 58.5 ± 12.60 | 0.426 | 0.021 |
| T.Chol (mg/dL) | 200.4 ± 43.88 | 209.1 ± 37.01 | 0.563 | 0.195 |
| sBP (mmHg) | 121.3 ± 8.49 | 122.8 ± 12.68 | 0.712 | 0.770 |
| dBP (mmHg) | 81.3 ± 9.01 | 75.3 ± 12.09 | 0.130 | 0.381 |
| PR (/min) | 79.5 ± 6.31 | 75.5 ± 8.32 | 0.156 | 0.552 |

Data are shown as Mean ± SD.

*P* value: One-way ANOVA was used

*P*^2^ value: *P* value from ANCOVA adjusted by age and sex

BW : body weight, BMI : body mass index, MR : metabolic rate, WC : waist circumference, FP, fat percentage, TBF : total body fat, FBS : fasting blood sugar, TG : triglyceride, HDL : high density lipoproteins, T. chol : total cholesterol, sBP : systolic blood pressure, dBP : diastolic blood pressure, PR : pulse rate.
