# Supplementary material for: Understanding the molecular aspects of oriental obesity pattern differentiation using DNA microarray
Source: J Transl Med. 2015 Oct 19;13:331. doi: 10.1186/s12967-015-0692-9 (PMC4617455; doi:10.1186/s12967-015-0692-9)
Supplement: Supplementary file 4 — 10.1186/s12967-015-0692-9 Clinical characteristics of 10 randomly selected subjects and 10 volunteers representing liver depression syndrome (LDS) pattern the samples of whom were used for the quantitative real-time PCR analyses of carcinoembryonic antigen-related cell adhesion molecule 3 (CEACAM3), cystein-serine-rich nuclear protein 1 (CSRNP1), and interleukin 8 receptor, alpha (CXCR1) genes. [file 12967_2015_692_MOESM4_ESM.docx]

**Supplementary Table 4. Clinical characteristics of 10 randomly selected subjects and 10 volunteers representing liver depression syndrome (LDS) pattern the samples of whom were used for the quantitative real-time PCR analyses of carcinoembryonic antigen-related cell adhesion molecule 3 (CEACAM3), cystein-serine-rich nuclear protein 1 (CSRNP1), and interleukin 8 receptor alpha (CXCR1) genes.**

|  | Random (n=10) | LDS (n=10) | *P* value | *P*^2^ value |
| --- | --- | --- | --- | --- |
| Gender | Male=2, Female=8 | Male=0, Female=10 |  |  |
| Age (yrs) | 35.4 ± 8.90 | 45.6 ± 8.36 | 0.017 |  |
| Height (cm) | 162.0 ± 6.18 | 155.9 ± 4.21 | 0.019 | 0.011 |
| BW (kg) | 75.7 ± 7.84 | 71.5 ± 9.31 | 0.297 | 0.381 |
| BMI (kg/m^2^) | 28.8 ± 2.27 | 29.0 ± 2.78 | 0.869 | 0.996 |
| MR (kcal/day) | 1343.0 ± 216.13 | 1490.0 ± 494.95 | 0.401 | 0.559 |
| WC (mm) | 98.0 ± 8.42 | 99.4 ± 8.56 | 0.703 | 0.926 |
| FP (%) | 37.5 ± 5.56 | 38.6 ± 5.63 | 0.654 | 0.677 |
| TBF (%) | 28.3 ± 4.87 | 27.8 ± 6.74 | 0.860 | 0.791 |
| FBS (mg/dL) | 94.4 ± 6.15 | 99.3 ± 5.89 | 0.085 | 0.064 |
| TG (mg/dL) | 100.0 ± 34.77 | 114.0 ± 57.79 | 0.520 | 0.729 |
| HDL (mg/dL) | 55.0 ± 13.10 | 53.3 ± 8.30 | 0.733 | 0.317 |
| T.Chol (mg/dL) | 203.5 ± 50.19 | 196.7 ± 43.49 | 0.750 | 0.577 |
| sBP (mmHg) | 120.7 ± 7.51 | 124.7 ± 13.15 | 0.415 | 0.429 |
| dBP (mmHg) | 79.1 ± 8.05 | 75.1 ± 11.41 | 0.377 | 0.619 |
| PR (/min) | 81.1 ± 7.49 | 80.9 ± 7.40 | 0.953 | 0.385 |

Data are shown as Mean ± SD.

*P* value: One-way ANOVA was used for cross-sectional comparison among three groups

*P*^2^ value: *P* value from ANCOVA adjusted by age and sex

BW : body weight, BMI : body mass index, MR : metabolic rate, WC : waist circumference, FP, fat percentage, TBF : total body fat, FBS : fasting blood sugar, TG : triglyceride, HDL : high density lipoproteins, T. chol : total cholesterol, sBP : systolic blood pressure, dBP : diastolic blood pressure, PR : pulse rate.
