# Supplementary figures and images for: Understanding the molecular aspects of oriental obesity pattern differentiation using DNA microarray
Source: J Transl Med. 2015 Oct 19;13:331. doi: 10.1186/s12967-015-0692-9 (PMC4617455; doi:10.1186/s12967-015-0692-9)

## Slide 1
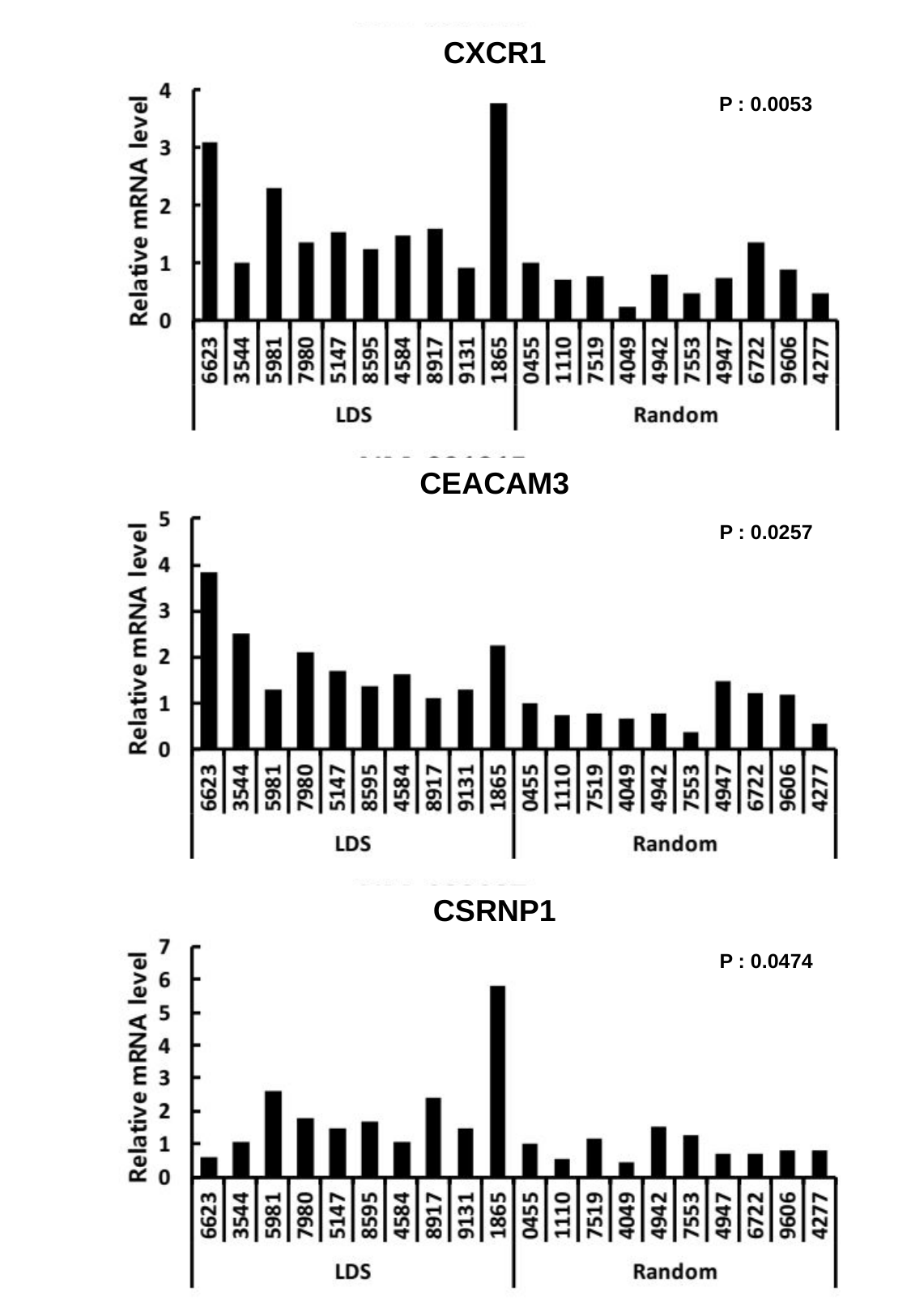

CXCR1
P : 0.0053
CEACAM3
P : 0.0257
CSRNP1
P : 0.0474

Supplement: Supplementary file 5 — 10.1186/s12967-015-0692-9 Expression levels of CEACAM3, CSRNP1, and CXCR1 genes in 10 randomly selected subjects (Random) or 10 volunteers representing LDS pattern (LDS) were measured using quantitative RT-PCR. P-value was determined using student’s t-test. [file 12967_2015_692_MOESM5_ESM.pptx]
